# Application of micro-dried droplets for quantitative analysis of particulate inorganic samples with LA-ICP-MS demonstrated on surface-modified nanoparticle TiO_2_ catalyst materials

**DOI:** 10.1007/s00604-020-04609-9

**Published:** 2020-11-05

**Authors:** Felix Horak, Andreas Nagl, Karin Föttinger, Andreas Limbeck

**Affiliations:** 1 Institute of Chemical Technologies and Analytics, TU Wien, Getreidemarkt 9/164-IAC, 1060 Vienna, Austria; 2 Institute of Materials Chemistry, TU Wien, Getreidemarkt 9/165, 1060 Vienna, Austria

**Keywords:** Laser ablation-ICP-MS, Quantitative calibration, Catalyst materials, TiO_2_, Surface-modified nanoparticles

## Abstract

A quick, flexible and reliable method was developed, based on laser ablation inductively coupled plasma mass spectrometry (LA-ICP-MS), for accurate assessment of nanomaterial composition with sample amounts in the picogram to nanogram range. We demonstrate its capabilities for the analysis of surface-modified TiO_2_ nanoparticulate (NP) catalyst materials. For sampling, suspensions of NP were deposited on a substrate material, ablated with a pulsed laser and then analysed using quadrupole ICP-MS. The calibration and quantification approach is based on the use of so-called micro-dried droplets (μDD) as the standard material. To overcome some of the major drawbacks of conventional dried droplet approaches, self-aliquoting wells were used in this work. By mimicking the ablation conditions for the sample and standard, it was possible to create a pseudo-matrix-matched calibration, not only for this specific NP composition but also for a larger variety of samples. A commercially available reference material (AUROlite™, Strem Chemicals) was used to compare the method against established methods such as slurry analysis and microwave-assisted digestion in combination with subsequent liquid sample measurement. The results obtained with the proposed procedure (0.74%wt ± 0.13%wt) are in good agreement to a certified value (0.8%wt) and added an additional layer of information. Due to the significantly reduced sampling size in comparison with the investigated liquid measurement approaches, it was possible to obtain information about the homogeneity of the catalyst material. The results indicate that the AUROlite™ reference material has a heterogeneous loading which requires more than 300 pg of material to be used to cancel out. This was not observed for the custom materials discussed in this work.

**Supplementary Information:**

The online version contains supplementary material available at 10.1007/s00604-020-04609-9.

**Figure:**
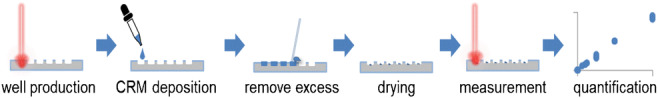
Graphical abstract

## Introduction

Titania is one of the most studied materials for photocatalysis [1] and also of high importance for supported metal nanoparticles in heterogeneous catalysts [2] due to its strong metal-supported interaction with noble metals. Ever since Haruta et al. [3, 4] reported the, rather surprising, high activity of gold nanoparticles in CO oxidation, gold received much attention as a catalyst material for a wide range of reactions in both liquid and gas phases, often with titania as the support material. The exact knowledge of the metal loading is crucial to assess the performance of a specific catalyst composition. Determining the number of active sites and thus the calculation of turnover frequencies (TOFs), a key figure in catalysis, relies on this knowledge. Thus, analytical methods for fast and reliable analysis are needed to improve material properties and to support the development of novel compositions.

The characterization of nanoparticle materials is usually performed with so-called bulk analysis methods such as X-ray fluorescence analysis with wavelength-dispersive or energy-dispersive (WD/ED) detection XRF or conventional liquid analysis as slurry [5–7] or after digestion [8, 9] using atomic absorption spectroscopy (AAS), inductively coupled plasma optical emission spectrometry (ICP-OES), or ICP-MS. These methods provide accurate and precise information about the average composition of the analysed material; however, information about the homogeneity of NP materials remains inaccessible with these established methods. Some information about the homogeneity of the individual composition can be derived via SEM/TEM-EDX analysis, but this requires specialized sample preparation and is tedious to evaluate. Analysis of NP agglomerates is also possible using secondary ion mass spectrometry (SIMS), which provides excellent sensitivity and allows investigations with a spatial resolution in the lower submicrometer range; however, quantitative analysis is difficult since SIMS is known to be affected by strong matrix effects [10].

In recent years, laser ablation inductively coupled plasma mass spectrometry (LA-ICP-MS) has proven to be a potent technique for the elemental analysis of small quantities such as single-micron-sized and smaller particles [11, 12] and similar non-bulk-like samples such as nanoparticulate suspensions [13, 14]. However, LA-ICP-MS measurements typically struggle with quantification and are therefore most commonly performed semi-quantitatively as an applicable reference material is barely available, especially for novel materials such as surface-modified nanoparticulates. Since the preparation and characterization of in-house standards is laborious and often challenging, an alternative strategy for the production of suitable standard materials is needed.

For this purpose, various approaches based on dried droplets [15–17] have been suggested in the past. Whilst side effects such as the “coffee-stain” effect and size discrimination [18, 19] due to increased evaporation rate towards the rim of the droplet could be overcome by complete consumption of the droplet and by addition of an internal standard [20], this procedure can be very time consuming and does not reflect the short transient nature of a particle ablation. Calibration procedures based on modified inkjet printers [21] are also reportedly capable of producing reliable calibration curves for small amounts of samples, though the process of spiking and mixing a specific target element composition can be tedious. To overcome the drawbacks associated with the use of dried droplets, recently, the use of micro-dried droplets (μDD) with diameters smaller than the applied laser beam has been introduced [22]. The use of micro-grooves represented an improvement of this approach as shown by Nischkauer for whole blood analysis [23] or Weiss et al. for the analysis of boride thin films [24]. The fundamental concept of the method is to provide a hydrophilic cavity on an else hydrophobic surface which is then filled with a specific amount of liquid reference solution. As the hydrophobic surface of the substrate is not likely to accumulate any residue from the liquid standard solution, the total amount of deposited material is governed by the dimensions of the hydrophilic wells.

Here, we present a further optimized variant of the μDD approach, which is not limited to the determination of elemental ratios, such as presented in earlier works, but also enables complete quantitative analysis for a large variety of elements. The optimized procedure was applied for the quantitative analysis of surface-modified catalyst materials, both commercially available (AUROlite™) and with a custom-designed composition. To assure the accuracy of the method, it was compared to established conventional elemental analysis techniques such as slurry-ICP-OES and liquid digestion followed by ICP-OES analysis of the investigated certified reference material.

## Experimental

### Chemicals, reagents and materials

p.a. grade ethanol, hydrochloric acid, hydrofluoric acid, hydrogen peroxide solution and nitric acid were purchased from Merck (Germany, https://www.merckmillipore.com) and diluted with purified water prepared by a Barnstead EASYPURE II system (Thermo Fisher Scientific, USA, https://www.fishersci.de) as required. Single-element standards were purchased from Merck (Si, Al and Gd), SPEX (https://www.spexcertiprep.com) (U), VWR (https://www.vwr.com) (Sr and Au), ROTH (https://www.carlroth.com) (Ti) and Alfa Aesar (https://www.alfa.com) (Os). Liquid standard solutions with varying compositions and concentrations were prepared just before use by dilution of the respective single-element standards.

AUROlite™ (Strem Chemicals, https://www.strem.com) was used as a certified reference composition. For the synthesis of the custom catalyst material, rutile (TP Hombikat Mikrorutil, Venator, https://www.americanelements.com), anatase (Sigma-Aldrich, https://www.sigmaaldrich.com), gold(III) chloride (Sigma-Aldrich) and urea (Merck) were used. Makrolon® (Bayer Material Science, https://www.covestro.com) was selected as the substrate material for the μDD production and the analysis of all solid samples.

### Instrumentation

A NWR213 laser ablation system (esi, http://www.nwrlasers.com) was used for both the preparation of the μDD wells and the respective analysis of particles and reference materials. For the LA-ICP-MS analysis, the NWR213 laser was coupled to a quadrupole ICP-MS (Thermo iCAP Qc, Thermo Fisher Scientific). The transfer line was modified to increase the washout time to allow more observations per analysis run. Determination of the depth and dimensions of individual wells was carried out using a DektakXT profilometer (BRUKER, https://www.bruker.com).

Samples were ultrasonicated with an ultrasonic cleaner USC 200 TH (VWR) and vortexed with a Vortex Genie 2 (Scientific Industries, https://www.scientificindustries.com).

Sample loading steps as well as loading of the μDD cavities were performed in a VFT 1525 ultraclean laminar flow hood (WEISS Technik, https://www.weiss-technik.com).

Microwave-assisted sample digestions were performed using an Anton Paar Multiwave 3000 (Anton Paar, https://www.anton-paar.com); liquid sample solutions were analysed using an iCAP 6500 ICP-OES spectrometer (Thermo Fisher Scientific) equipped with a CETAC ASX-520 autosampler (CETAC Technologies, http://www.teledynecetac.com).

A FEI Quanta 250 FEGSEM (https://www.fei.com) was used for the SEM measurements.

(S)TEM imaging was performed with a FEI Tecnai F20 FEG-TEM instrument.

### Synthesis and characterization of custom-modified catalyst particles

**Fig. 1:**
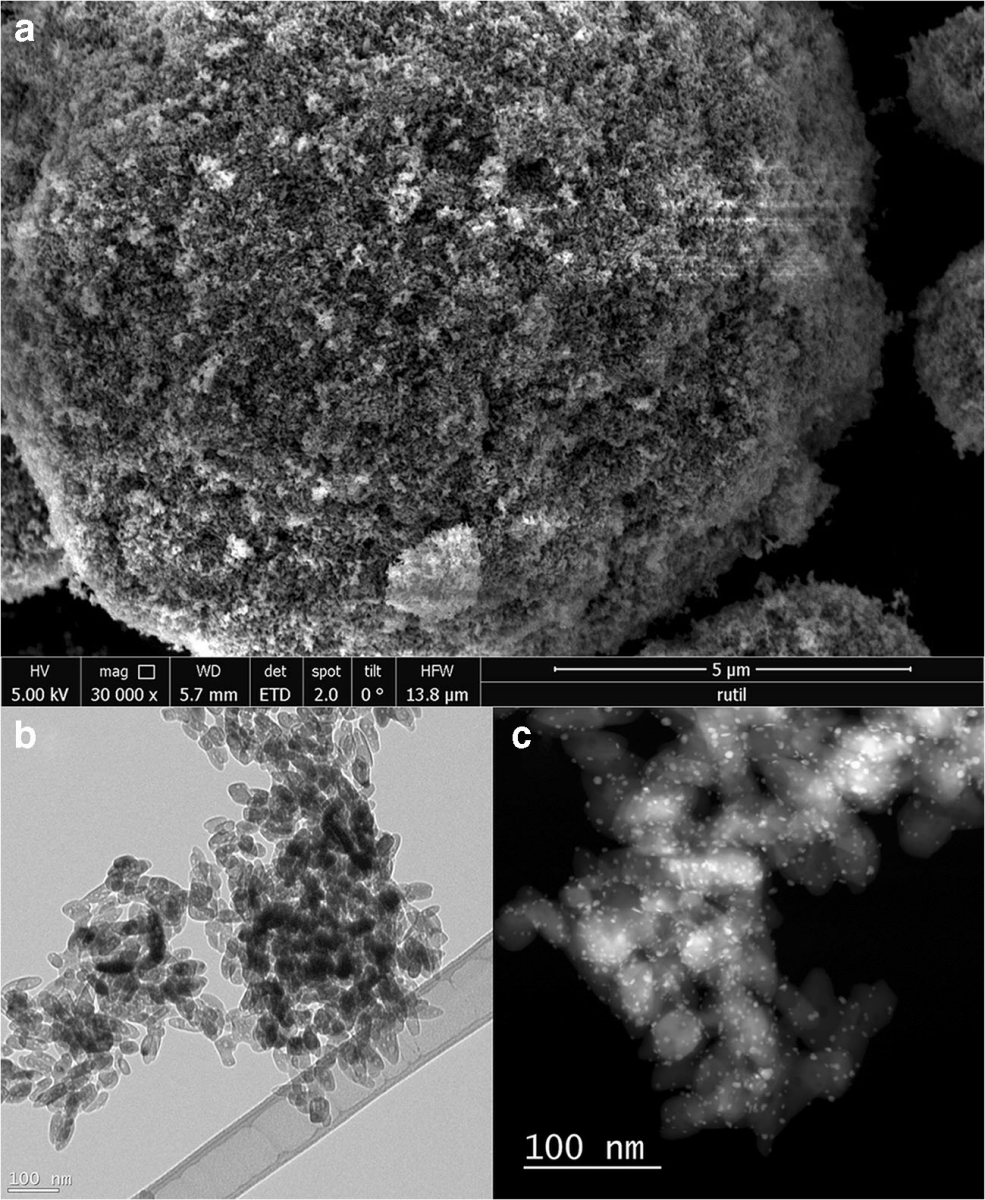
Electron microscopic images. **a** SEM image of TiO_2_ clusters. **b** TEM micrograph of TiO_2_ clusters. **c** STEM-HAADF image of the finished catalyst material with deposited Au NP. The samples were imaged after the pretreatment process of the catalyst (oxidative treatment in 20% O_2_ at 400 °C followed by reductive treatment in 5% H_2_ at 300 °C in a continuous-flow fixed-bed quartz reactor under atmospheric pressure) on Lacey carbon grids

Preparation of the Au nanoparticle catalysts supported on TiO_2_ was done following a well-established recipe by Zanella et al. [25, 26], which is known to lead to small well-dispersed particles (synthesis recipe is provided in ESM). Three different TiO_2_ supports were employed: rutile, anatase and a physical mixture of 20% rutile and 80% anatase. The individual particles tend to agglomerate into larger particles even in the highly diluted solutions used for the SEM and TEM images, as can be observed in Fig. 1, which shows various electron microscopic images of the larger titania NP (Fig. 1a and b) and the smaller Au catalyst NP on top (Fig. 1c). For the SEM measurement, a Schottky emitter with an acceleration voltage of 5.00 kV was applied. The working distance (WD) for the measurements was 5.7 nm and an Everhart-Thornley detector (ETD) was used. Due to the limited resolution of the SEM instrument, individual titania NP cannot be resolved and only larger clusters can be observed, and this is even more true for the smaller Au NP. (S)TEM imaging allows analysis of individual particles. The images were made with an acceleration voltage of 200 kV. The Au NP which forms during the pretreatment can be observed as bright spots in the TEM image (Fig. 1c). The average size of the deposited Au NP was determined to be 3.3 ± 1 nm by STEM-HAADF with a sample size of *n* = 292 particles.

### Production of μDD (theory and instrumentation)

Using a NWR213 ns laser, multiple sets of 100 self-aliquoting wells with a diameter of 50 μm were shot in 2-mm-thick PC (Makrolon®) plates. This substrate provides a sufficiently hydrophobic surface and is commercially available with high purity. The length and width of the substrate were freely adjusted to the available space in the ablation cell in the laser. For ease of handling, the slides used in this work were typically cut to match a typical microscope slide (75 mm × 26 mm). The typical layout of a μDD array consists of 20 lines with 5 wells resulting in a total amount of 100 wells per array. Within such an array 300 μm of distance was left between wells and 5 mm between arrays of varying concentrations. The wells were shot with a fluence of 1 J/cm^2^ and 80 shots per location. For consistent performance, a 3-s warm-up time was applied for each ablation and the ablation chamber was flushed with 200 ml/min of He gas. Laser warm-up was performed only at the beginning of an array. The warm-up duration was chosen to provide similar conditions for each position of the well rather than full power as the ablation on each position was shorter than the time required by the laser to reach energy-stable operation on the flash lamp–pumped laser.

After production of the wells, the slides were transferred to a vial with ultrapure H_2_O (Milli-Q, 18.2 MΩ cm) and ultrasonicated for 15 min to remove debris from the surface.

Afterwards, the slide was removed from the vial and excess water eliminated with pressurized air, before transferring the slides to a laminar flow hood for the loading of the sample.

For loading, 10 μl of liquid CRM (concentration range ng/ml–μg/ml) was positioned near a well-array and subsequently pushed over the full length of the array, using a clean slide of a small PC slide. To guarantee homogeneous loading, the diameter of the droplet should be approx. 30% larger than the dimensions of the well-array. As only a fraction of the originally deposited droplet is used up to fill the μDD wells, excess liquid must be wiped off the slide or blown away with pressurized air.

**Fig. 2:**
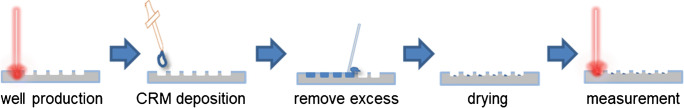
Schematic μDD production from production of the individual wells with a pulsed laser followed by analyte deposition and filling of the wells and ending in analysis via laser ablation

The small amount of liquid within the wells evaporates within seconds; therefore, the μDD array is ready to use immediately after the loading step without the need for additional drying time. A shortened schematic overview of the complete procedure can be seen in Fig. 2.

### Sample preparation for LA-ICP-MS analysis

Prior to sampling, the source material is thoroughly homogenized via shaking and stirring. A small amount of particles (approx. 1 mg) is then transferred to a vial containing 1 ml ethanol (99%, p.a.). The suspension is then thoroughly mixed by vortexing or ultrasonication, keeping in mind that vortexing does little to destroy agglomerates and ultrasonication may result in loss of surface modifications due to cavitation. The high difference in density results in particles quickly accumulating towards the bottom of the suspension. Therefore, a strict guideline for sampling is mandatory. After 30 s of vortexing, the suspension is ultrasonicated for additional 30 s. A subsample of 200 μl is taken from approx. 1 cm above the bottom of the suspension within 1 min of ultrasonication and transferred on a Makrolon substrate slide. Different particle suspensions varying in compositions or concentrations were deposited on the same substrate slide when there was enough space.

Effects influencing the size variation over the dimensions of the dried solution, such as the coffee ring effect, can be reduced by speeding up the evaporation of the ethanol by heating the slide to 70 °C. Nevertheless, during evaporation of the dispersing agent, agglomeration of the nanoparticles was observed, resulting in the formation of NP clusters. LA-ICP-MS analysis of the produced NP samples can be performed without any further treatment steps; in particular the particles do not require additional fixation for measurement.

As the resolution of the optical observation system in most laser ablation instruments is not capable to reliably identify single particles of less than 1-μm diameter, this method is optimized for the analysis of small agglomerates of NP. However, by adjusting the particle load, single-particle analysis can also be reliably achieved for larger particles. For the evaluation of this method, we selected the commercially available reference material AUROlite™ (Strem Chemicals).

### Measurement conditions

The instrument settings were selected for best signal to noise (S/N) ratio and fast response to ensure sufficient data points on short transient signals. In contrast to bulk analysis, the measurement of a finite small amount such as small NP clusters does not allow for continuous stable conditions. Instead, a high and short transient signal is observed. Therefore, a compromise between high signal intensity and sufficient observation points was achieved with the conditions outlined in Table 1. For both μDD and NP cluster analysis, the same ablation conditions were applied. Using a larger diameter in comparison to the dimensions of the μDD well, it was possible to ensure complete consumption of the deposited material within only a few laser pulses and thus a very fast washout, similar to the signal response of a particle analysis. Using the same diameter for particle analysis ensures equal amounts of substrate introduced into the plasma. As most of the material which is introduced into the plasma, thus governing the plasma conditions, is the sample substrate for both the μDD well and the nanoparticle measurement, the μDD calibration approach can be classified as “pseudo-matrix-matched”.

**Table 1:** Instrument settings for the LA-ICP-MS analysis of μDD and TiO_2_ NP clusters

ICP-MS parameters	
Plasma power	1400 W
Nebulizer flow (Ar)	0.9 l/min
Carrier flow (He)	0.85 l/min
Auxiliary gas	0.8 l/min
Cool gas	14 l/min
Dwell time	7 ms
Observed isotopes	^49^Ti, ^197^Au
LA parameters	
Frequency	20 Hz
Laser diameter	80 μm
Laser fluence	6 J/cm^2^
Laser warm-up	8 s
Shots per location	40
Ablation mode	Spot ablation

### Data treatment

The analysis of a short transient signal, such as produced by ablation of a small finite amount of material, is always a trade-off between sensitivity and precision. Fast washout cells have been reported to significantly improve the overall sensitivity for LA-ICP-MS measurements. However, in peak-hopping analysers such as quadrupole or scanning sector field mass analysers, a short transient signal limits the amount of elements which can be observed during each measurement. Therefore, a consensus between overall signal intensity and duration has to be reached. Preliminary experiments revealed optimum results using a dwell time of 7 ms and a limitation to two isotopes. In this work, a threshold of 25 cycles with more than 24 events per analysis cycle for each observed element is applied. Measurements with lower count rates or cycle count were discarded for the evaluation of the composition for each material. The measurements were then evaluated based on a robust estimation approach as presented by Walsh et al. [27]. For quantitative measurements, these requirements are expanded to the absence of spikes in the transient signal. Whilst some effort was made in the LA-ICP-MS community for both detection and elimination of spikes in transient signals, complete rejection of the measurement is the only way to ensure overall data integrity. The mean of the first 50 cycles of each measurement was used for blank correction as the substrate does not introduce any background signal for the observed *m*/*z*.

### Reference measurement for verification of results

Whilst there are a few agreed-upon guidelines for the analysis of TiO_2_ nanoparticles (i.e., The American Society for Testing and Materials guidelines ASTM D476 – 15 and ASTM D1394 - 76), these are typically for off-the-shelf applications where only information about the size, quantity or water content of TiO_2_ is required. Especially for non-off-the-shelf compositions, where information about varying (surface) modifications is needed, the amount of reference guidelines is very limited as various precious metals require different digestion approaches. The chemical and thermodynamical stability of nanoparticulate TiO_2_, whilst making the material so highly sought after, is a challenging matrix for conventional analysis methods. In this work, two conventional liquid ICP-OES analysis methods were applied.

### Slurry analysis

In slurry procedures, particulate samples are not digested with mineral acids but dispersed in suitable liquids. This method is especially useful for nanoparticulate samples which are difficult to dissolve and remain suspended long enough for the measurement. A major disadvantage of this method is the possibility of particle agglomeration as well as sedimentation of particles during the measurement, which cannot be easily corrected by addition of an internal standard to the solution.

For the slurry measurement, 15 mg of AUROlite™ was dispersed in 10 ml 2% (vol/vol) HNO_3_ and ultrasonicated for 15 min. From this dispersion, subsamples of 250 μl and 2000 μl were further diluted to 15 ml in 2% HNO_3_ with Europium as the internal standard two times each. Each sample was vortexed for 2 min prior to the ICP-OES analysis. To minimize the risk of clogging, a high-solid nebulizer was used in conjunction with a quartz spray chamber. The complete list of measurement conditions applied in the ICP-OES measurements is shown in Supplementary Table 1.

### Microwave digestion

For each replicate, a subsample of 15-mg particles underwent a two-step microwave digestion. For the first digestion, a mixture of 4 ml HNO_3_ (conc.), 1 ml H_2_O_2_ (conc.) and 1 ml HF (conc.) was added to the particles and microwave-digested for 30 min at a maximum temperature of 180 °C and a maximum pressure of 20 bar. Afterwards, the samples were cooled to room temperature and prepared for an additional digestion run. In the second digestion setup, 2 ml HCl (conc.) and 0.66 ml HNO_3_ (conc.) were added to the vials and digested for further 30 min using the same conditions.

The derived clear sample solutions are then diluted by a factor of 1000 with 2% HNO_3_ (vol/vol) for subsequent ICP-OES analysis. The conditions for the analysis were the same as those for the slurry measurement (see Supplementary Table 1).

## Results and discussion

### Verification of total amounts in a μDD well

Before using the μDD as the calibration standard for subsequent analysis, the total amount retained in the micro wells must be determined. Profilometric means of determining the volume of each well are biased by the roundness of the stylus and therefore possibly not sufficient for calculating the amount of analyte deposited per well (see Supplementary Figure 1) and visual methods are hindered by the low contrast of the transparent Makrolon. Therefore, the total amount per well was determined by an analysis scheme based on overnight leaching of analyte-filled μDD arrays (see Supplementary Figure 2). For this, a fixed amount of wells (50) was loaded with solutions containing varying analyte concentrations (25, 50, 100 and 200 μg/ml in 2% HCl). Blanks were prepared by the application of only diluted acid.

The areas around the wells were then cut from the sheet and discarded, whereas the part containing the wells was transferred into a centrifugation vial with 1 ml 2% HCl (vol/vol) and ultrasonicated for 15 min. The wells were left to leach overnight at room temperature and vortexed briefly before measuring. The concentration of the leached amount was determined by means of liquid mode ICP-MS with In as the internal standard (see Supplementary Table 2 for the instrumental settings). Between the analyte contents of the applied liquid reference materials and the measured concentrations of the leachates, an excellent linear correlation was observed, enabling the calculation of the deposited mass of analyte per well. The typical error of these measurements was around 5% RSD.

**Fig. 3:**
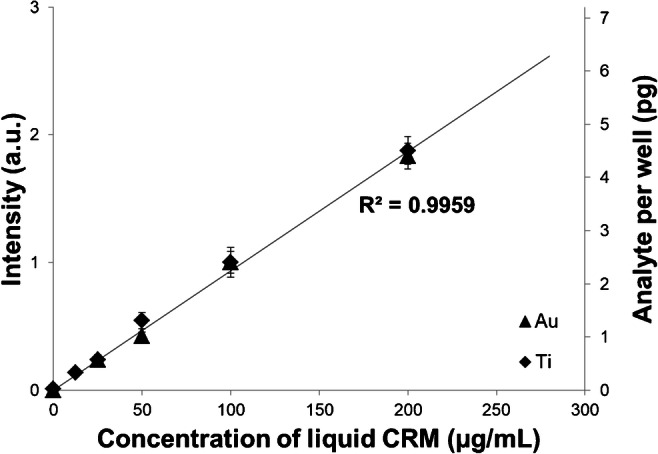
Normalized calibration plot for Au and Ti (*n* = 20, 2*σ*). Quantitative amounts are based on leaching experiments

The correlation between analyte concentration and total amount per well was verified for multiple elements, which all showed a linear behaviour and followed the same slope (see Supplementary Figure 3 for the leaching calibration plot). Figure 3 shows a normalized calibration plot for Au and Ti, of which a version with more elements is presented in Supplementary Figure 4. These findings indicate that the amount of deposited material is governed by the well dimensions and analyte concentration in the original droplet and is not element specific.

### LA-ICP-MS analysis of μDD well standards

**Fig. 4:**
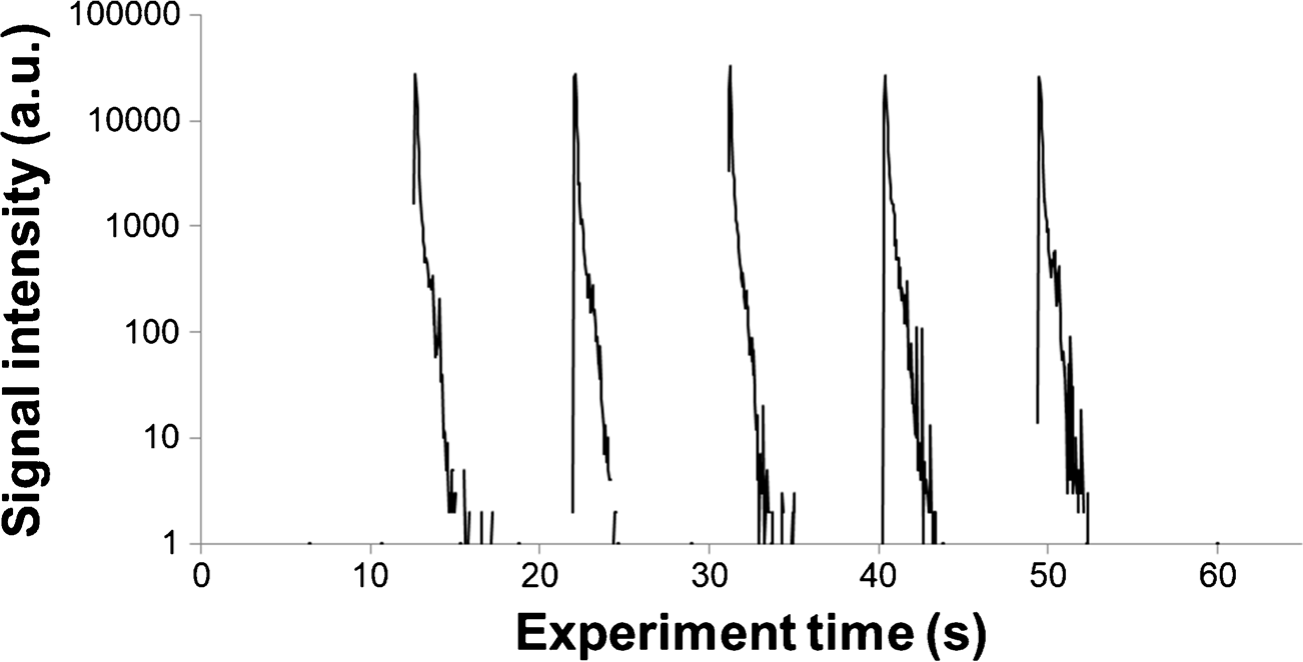
Line scan over multiple (5) μDD wells with 200 mg/g analyte, indicating no material deposition between wells

For this measurement approach, it is crucial to verify the absence of deposits from the original droplet outside of the μDD array to provide valid results. This was verified by performing LA-ICP-MS line scans over the surface of the slide in the original path of the droplet. As expected, the μDD wells showed huge signals for the elements of interest; however, outside the wells, some residual deposits have been found at surface defects like scratches and dust particles, as well as the rim of the slide where the droplet was pushed over after the loading. To eliminate these unintentional contributions, loading of the slides and all further treatment steps were performed in a laminar flow hood to reduce the risk of particle depositions from the lab atmosphere on the surface. Additionally, the edges of the slides were wiped clean with saturated alcohol wipes prior to LA-ICP-MS analysis. These two rather simple measures provided sufficient “decontamination” for these analytes, as no signal was measured outside of the wells in all subsequent experiments. As can be observed in Fig. 4, which shows a line scan over 5 wells in a μDD array, no signal originating from the analyte can be detected aside from the μDD. The peak width of derived LA-ICP-MS signals is determined by the speed of the stage movement and the washout performance of the laser ablation system.

The linear range of the calibration depends on availability and solubility of the desired analytes, but typically covers 4–6 orders of magnitude. Due to the choice of substrate and standards, the limits of detection for the method are in the range of attograms to femtograms, which depend mostly on the selected criteria for data evaluation, as outlined above. The minimum amount of signal necessary to be considered was already higher than 10 times the SD of the blank; therefore, the LOD/LOQ values were determined via the regression of the slope as this yielded a more conservative estimate. The limit of detection, derived this way, was 1.0 fg for Ti and 1.3 fg for Au.

### Analysis of AUROlite™ reference material

**Fig. 5:**
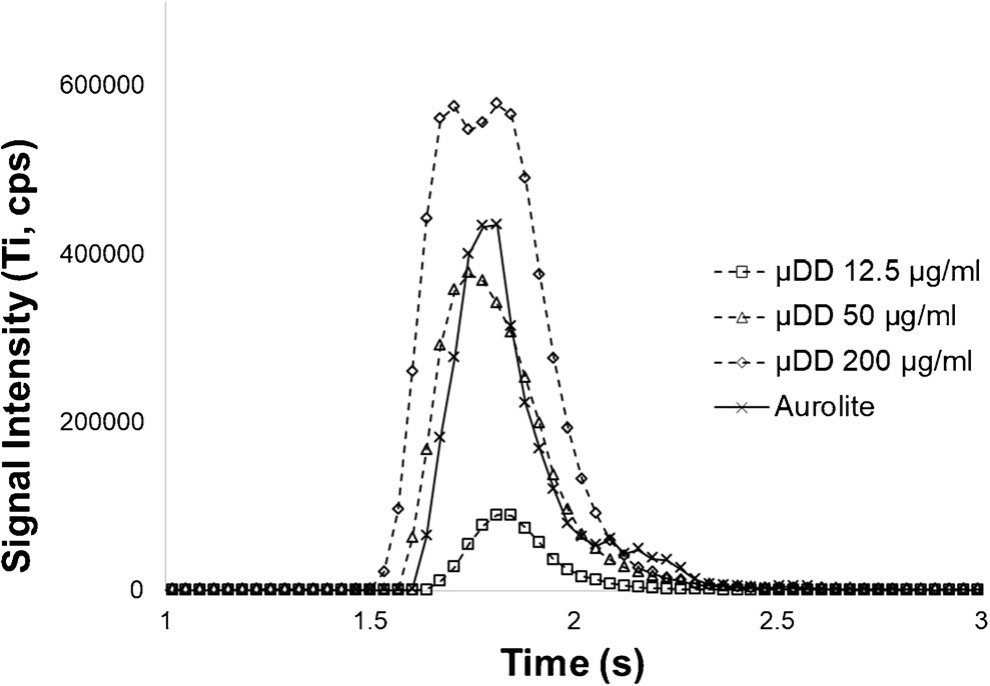
Comparison of transient signals from μDD and NP clusters; timescale modified for presentation purposes. The transit line was modified to broaden the transient signal to provide more data points per analysis run

For the LA-ICP-MS analysis, a suspension of AUROlite™ in ethanol was dispersed on Makrolon® and transferred to the laser chamber. For the selection of clusters to be analysed, it is important to only select clusters which are isolated enough from others to be ablated with the same beam diameter as the μDD, to establish similar measurement conditions. Therefore, only clusters which were barely visible in the optical microscope of the laser and well separated from other visible clusters were analysed. When comparing the transient signals and the overall intensities of the respective signals for μDD and AUROlite™ NP clusters (see Fig. 5), an overall good agreement in the signal shape of the standard material and sample can be observed. The Ti and Au signals observed during laser ablation of individual NP clusters were converted into absolute amounts using the determined calibration functions. This data evaluation approach leads to only one single data point for each NP cluster, since replicate measurements of the same cluster were not possible. In total, a number of 20 NP clusters were analysed, and 17 of these clusters were considered for evaluation of which the smallest amount exceeded the limit of quantification for Ti (3.0 fg) and Au (3.9 fg) by approx. a factor 50.

The reference value for %wt Au in AUROlite™ particles is 0.8%wt, with no stated uncertainty, whilst the microwave-assisted digestion and LA-ICP-MS measurement succeeded in reaching the same conclusion. With 0.77%wt ± 0.05%wt for the digestion and 0.74%wt ± 0.13%wt for the LA-ICP-MS measurement, respectively, both equally overlap with the certified value. The μDD method shows good agreement to the microwave digestion with a moderate decrease in precision. This outcome demonstrates the applicability of the developed LA-ICP-MS procedure. The slurry method resulted in a significant over-estimation at 1.5%wt ± 0.2%wt and did not agree with the certified value. This can be explained by erosion of the surface modification due to the acidic solution or due to cavitation during the ultrasonication step. Furthermore, the slurry method, whilst seemingly ideal as it does not require digestion of the sample, struggled with sedimentation and especially agglomeration of the samples leading to unpredictable intensities during measurements as well as frequent clogging of the nebulizer.

**Fig. 6:**
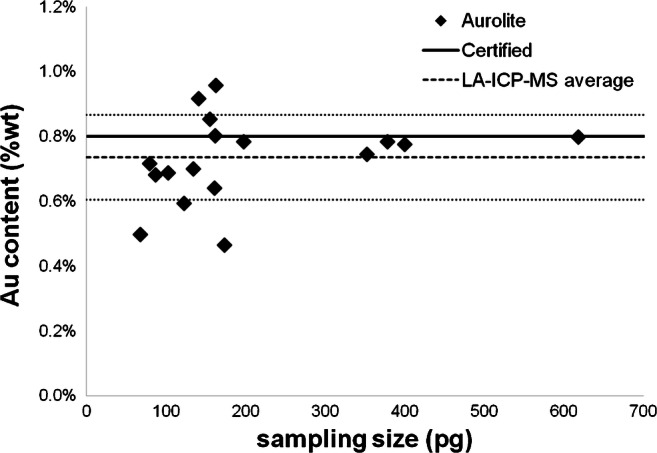
The determined Au content of the AUROlite material in correlation to the sampling size. When analysing less than 200 pg, significant variation in the determined Au content can be observed

Compared to the two reference methods for assessment of the gold content, which provide only bulk information, the LA-ICP-MS procedure also hints about the material homogeneity. As can be deduced from Fig. 6, which presents the dependence of the determined Au/Ti ratio from the analysed sample mass, the composition of the AUROlite™ reference material is inhomogeneous. Results observed for the investigated NP clusters provide no information about measurement uncertainty due to the integration of whole peak intensities and complete consumption of the material in one measurement, resulting in only one single data point per LA-ICP-MS analysis. When larger clusters and thus higher amounts of AUROlite™ were ablated, an excellent agreement with the certified composition was found. However, for experiments with lower sample intakes, a distinct deviation of the achieved Au/Ti ratios from the target value was observed. Problems in the quality of LA-ICP-MS analysis could be excluded as a possible reason for this outcome, since the Au and Ti signals recorded in these measurements were still orders of magnitude above the respective limits of quantification and the analysis of reference μDD with similar amounts does not suffer from similar deviation as can be seen in Supplementary Figure 5 (approx. 5% RSD). Hence, the deviation from the certified value indicates that the homogeneity of the AUROlite™ material is limited when looking towards sampling sizes of less than 300 pg per measurement (which corresponds to amounts of less than 3 pg Au).

### Analysis of custom-designed surface-modified TiO_2_ NP

**Fig. 7:**
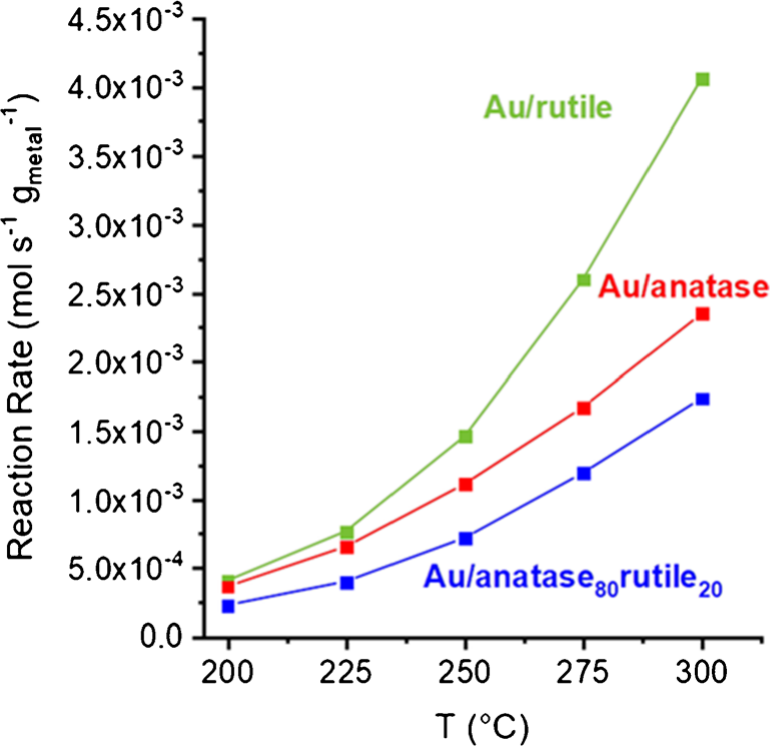
Reaction rate of the individual catalyst materials normalized to the determined Au content, in regard to reaction temperature

Reaction rates and turnover frequencies (TOFs) are essential characteristics of a catalyst. For calculating reaction rates and relating them to the amount of catalytically active metal nanoparticles, exact knowledge of the respective metal loading of the catalyst materials is essential. However, due to the application of different synthesis routes and sometimes occurring variations in the quality of synthesis, information about the exact loading is often not known. In this work, three Au catalysts on different NP support materials (anatase, rutile and an 80/20 blend of anatase and rutile) were synthesized in the same way and then analysed to evaluate their respective Au loading. In contrast to the previously described analysis of the AUROlite™ reference material, no significant dependence of the determined composition from the analysed sample size was found. A comparison of 20 individual clusters for each composition is available in Supplementary Figure 6. Whilst the rutile and anatase substrate both deposited similar amounts of Au (rutile 2.9%wt ± 0.12%wt, anatase 2.8%wt ± 0.17%wt), the 80/20 blend incorporated almost 0.5% more Au onto its surface (3.4%wt ± 0.24%wt). Figure 7 depicts the reaction rate per gram Au loading, as determined above, for the gas phase oxidation of ethanol to acetaldehyde in a fixed bed flow reactor. The exact quantification of Au on the catalyst surface allows the clear conclusion that Au/rutile is the most active support and a mixture of anatase and rutile is less beneficial than anatase. Despite the higher Au loading of the 80/20 blended support, this catalyst shows lower activity, which might attributed to the heterogeneity of the Au dispersion.

## Conclusions and outlook

In this work, it was demonstrated that the combination of liquid CRM with self-aliquoting micro-wells can be used for quantification in LA-ICP-MS with sample sizes in the picogram range. The absolute amount of analyte required for quantitative analysis is about 3.0 fg for Ti and 3.9 fg for Au and thus significantly reduced when compared to wet chemical approaches used for conventional bulk measurements. Thus, sampling of microgram subsamples during synthesis can be easily used for at-line quality control of a NP synthesis, as the samples require little handling aside from dispersion in alcohol and subsequent pipetting on a suitable substrate. This advantage is of special importance for analysis of valuable samples such as the investigated catalyst material containing expensive noble metals.

Applicability of the proposed procedure has been demonstrated by analysis of AUROlite™ reference material, indicating that the homogeneity of the material is limited for very small sampling amounts and the certified value is only achieved when more than approximately 300 pg is used for analysis. This observation agrees with TEM observations made prior to this work [28]. For smaller amounts, increased differences to the target value were observed, which did not originate from errors in the LA-ICP-MS analysis. Thus, the presented LA-ICP-MS provides additional information about the sample homogeneity which is not accessible with conventional digestion or slurry procedures. However, the precision of LA-ICP-MS analysis of a subset of picogram to nanogram clusters cannot (yet) compete with bulk measurement procedures.

The ease of μDD production and high flexibility in terms of composition have to be considered as the major advantage of this procedure, as it allows simple adjustment to the needs of the actual task, a prerequisite for the characterization of novel composite materials.

Another major benefit of the developed approach is the possibility of sample storage. Individual μDD reference standards have shown no significant amount of deterioration over the timeframe of multiple months and batch-to-batch variation can be minimized by well-defined operation procedures. Long-term stability can be further improved by covering the substrate surface with a thin layer of polyimide or similar polymers; thus, the possibility of sample contamination or the introduction of interferences is reduced.

## Supplementary Information

ESM 1 (PDF 1.34 mb)
