# Supplementary material for: Application of micro-dried droplets for quantitative analysis of particulate inorganic samples with LA-ICP-MS demonstrated on surface-modified nanoparticle TiO2 catalyst materials
Source: Mikrochim Acta. 2020 Nov 5;187(12):641. doi: 10.1007/s00604-020-04609-9 (PMC7644544; doi:10.1007/s00604-020-04609-9)
Supplement: Supplementary file 1 — (PDF 1.34 mb) [file 604_2020_4609_MOESM1_ESM.pdf]

Electronic Supporting Material

Application of micro-dried droplets for quantitative analysis of particulate inorganic samples with LA-ICP-MS demonstrated on surface-modified nanoparticle TiO_2 catalyst materials

Felix Horak[1], Andreas Nagl[2], Karin Föttinger[2], Andreas Limbeck[1]

[1] Institute of Chemical Technologies and Analytics, TU Wien, Getreidemarkt 9/164-IAC, Vienna, Austria

[2] Institute of Materials Chemistry, TU Wien, Getreidemarkt 9/165, 1060 Vienna - Austria

Nanoparticle Synthesis

The Au catalysts were synthesized by deposition-precipitation with urea with a nominal Au loading of 5%wt. The support oxide was added to an aqueous solution of $HAuCl_4$ and urea under stirring at 80°C for 4 h, which resulted in an orange precipitate. Afterwards, the catalyst was washed 5x with deionized water to remove chlorides and dried at 100°C. The materials were stored in the dark. The analysis of the catalyst materials was performed in this state, whereas for catalytic use, a so-called pretreatment step is performed (oxidative treatment in 20% O_2 at 400°C followed by reductive treatment in 5% H_2 at 300°C) in which the gold nanoparticles are formed.

Supplementary Table 1 Instrument settings for all ICP-OES measurements

Power	1200 W
Observation position	Radial
Observation height	5 mm
Nebulizer flow	1.2 L/min
Auxiliary gas	0.8 L/min
Cool gas	14 L/min
Observed emission lines	Ti: 334.941 nm (II), 323.452 nm (II), 338.376 nm (II)<br>Au: 242.795 nm (I), 267.595 nm (I), 208.209 nm (II)<br>Eu: 381.967 nm (II), 281.394 nm (II)

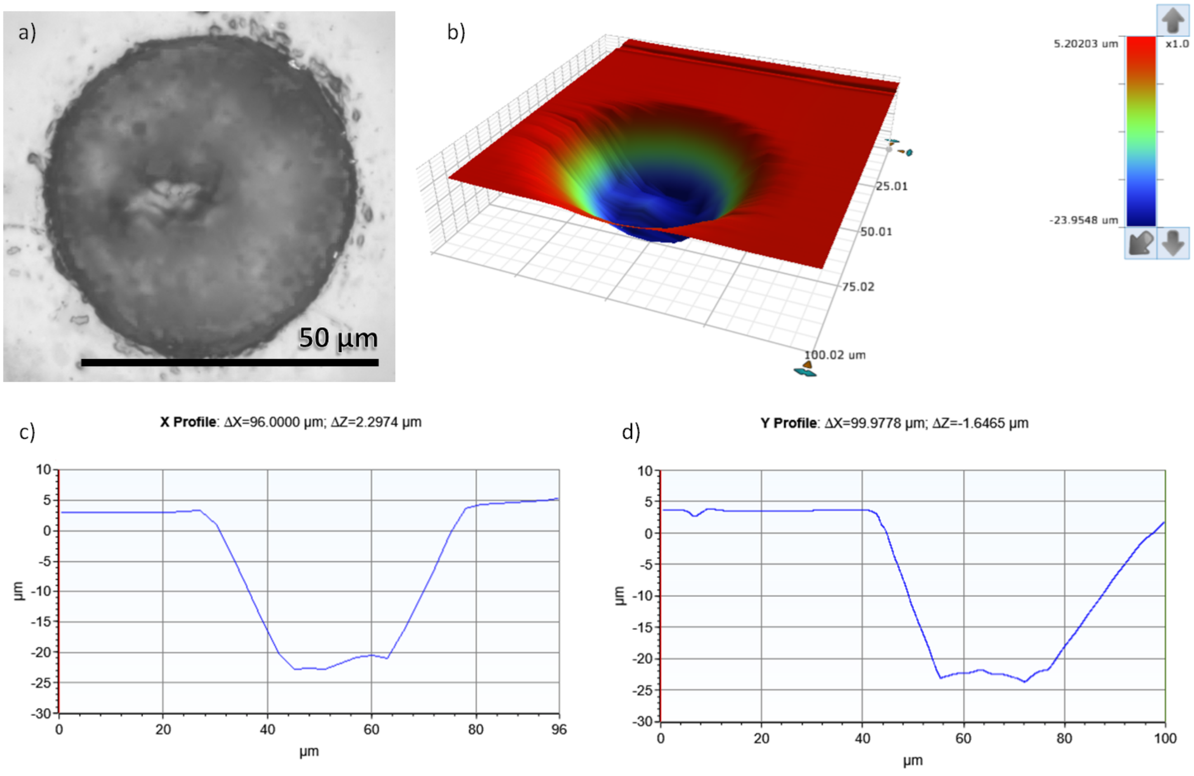

Supplementary Figure 1 visual and profilometric analysis of a single µDD well; a) shows a LF microscope image of a single µDD well, b) 3D plot over a well, c) and d) show a cross-section through the mapped well in X- and Y- orientation respectively.

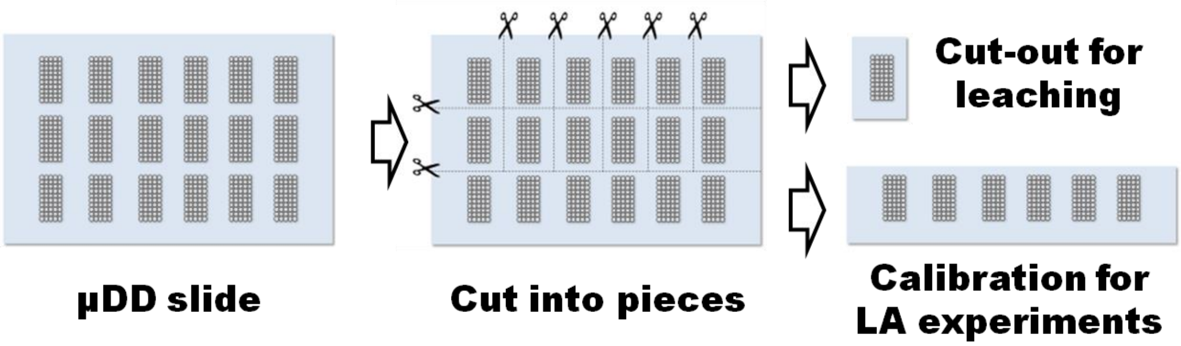

Supplementary Figure 2 schematic overview of the sample preparation for leaching and subsequent calibrations

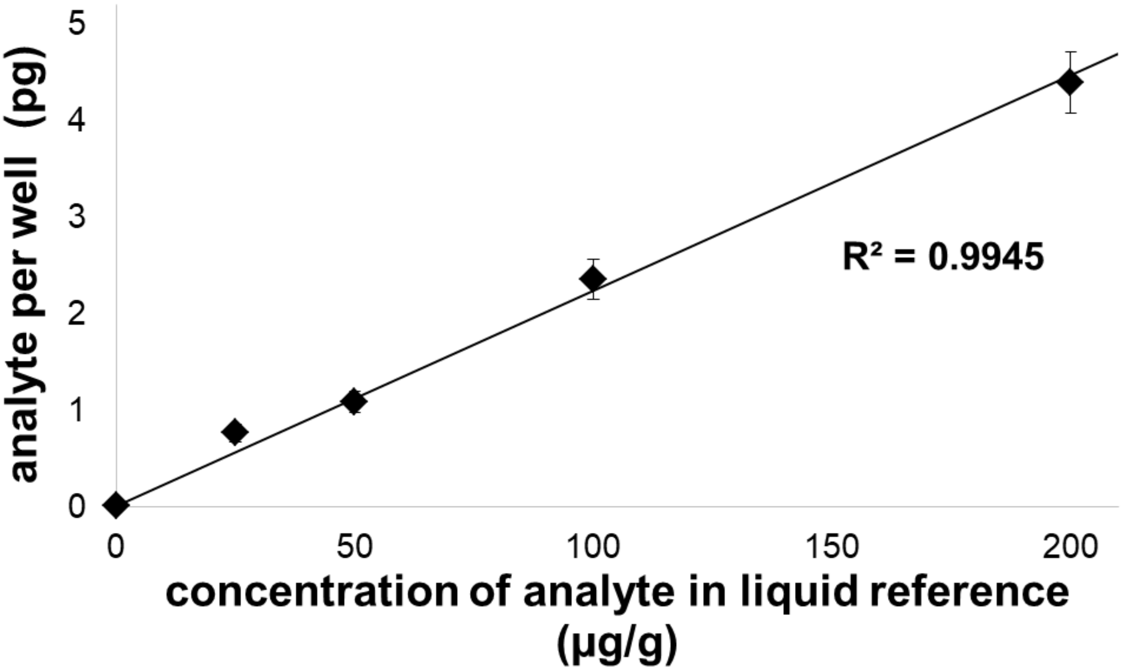

Supplementary Figure 3 combined calibration plot for leeching experiments carried out with Sr, Os and U (n = 6, 2σ).

Supplementary Table 2 Instrument settings for liquid ICP-MS determination of the analyte concentration in the leeched µDD wells.

Power	1400 W
Dwell time	20 ms
Observed Isotopes	^{87}Sr, ^{88}Sr, ^{113}In, ^{197}Au, ^{190}Os, ^{192}Os, ^{235}U, ^{238}U
Nebulizer flow	0.90 L/min
Auxiliary gas	0.8 L/min
Cool gas	14 L/min

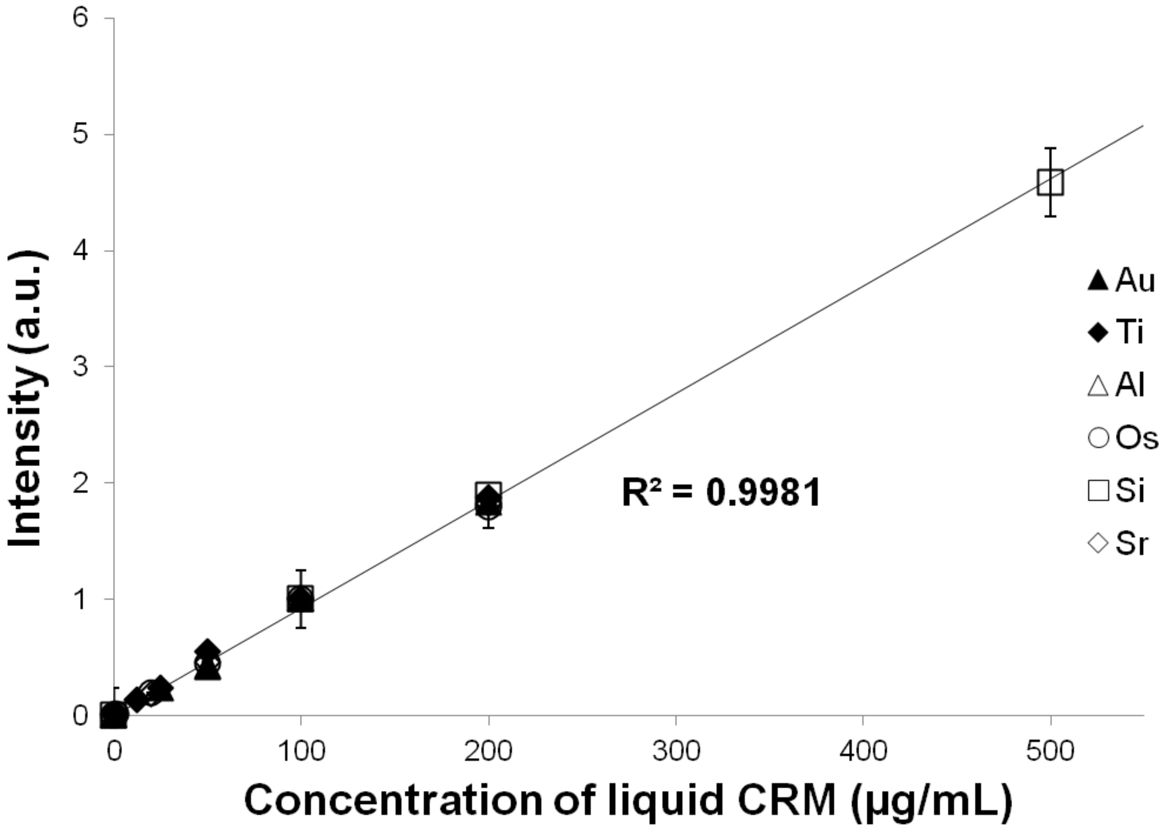

Supplementary Figure 4 Normalized calibration plot for ^{197}Au, ^{49}Ti, ^{27}Al, ^{192}Os, ^{29}Si and ^{88}Sr (n = 20, 2σ)

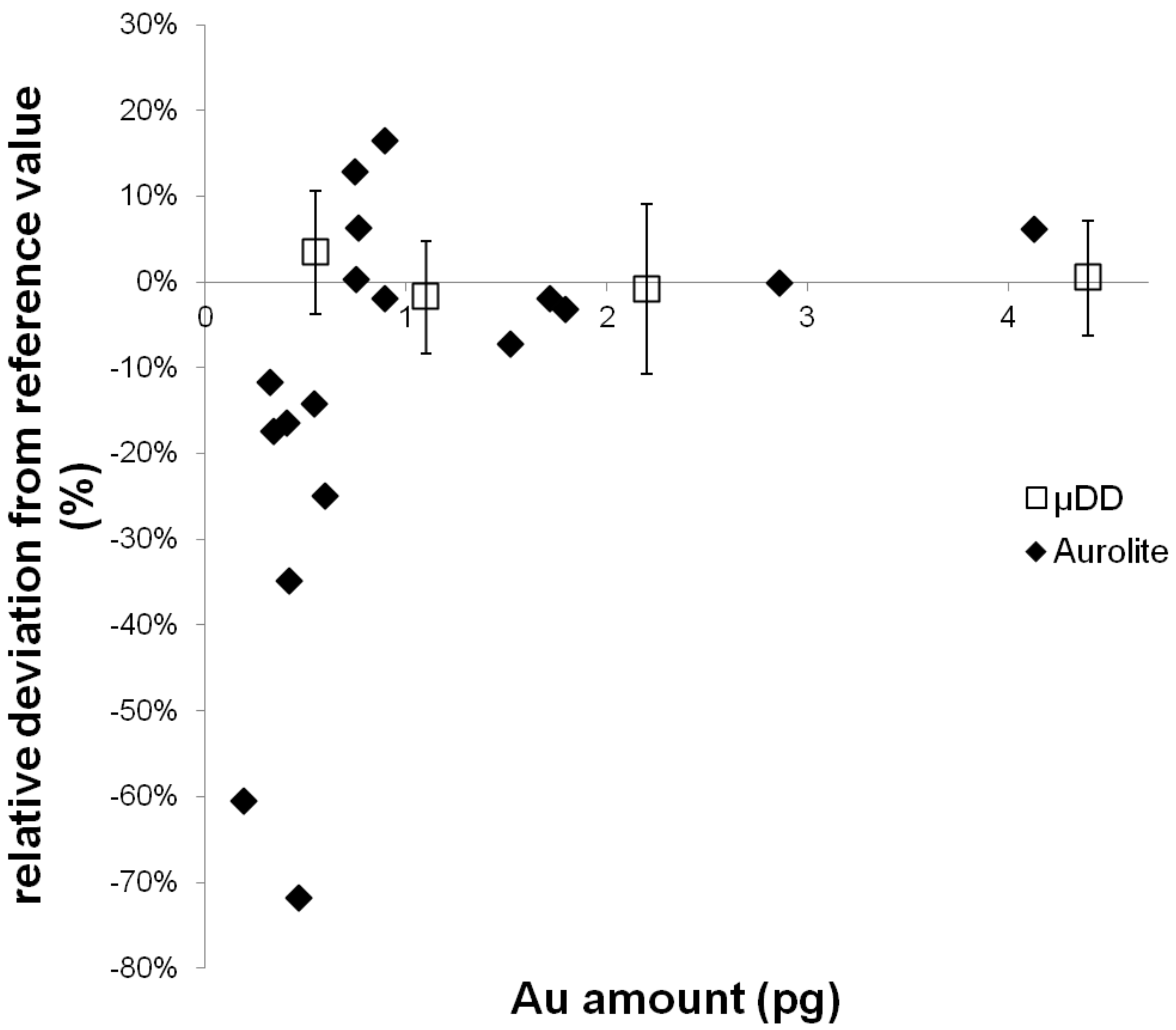

Supplementary Figure 5 relative deviation from reference value for Aurolite clusters and µDD reference material (n = 20, 2σ)

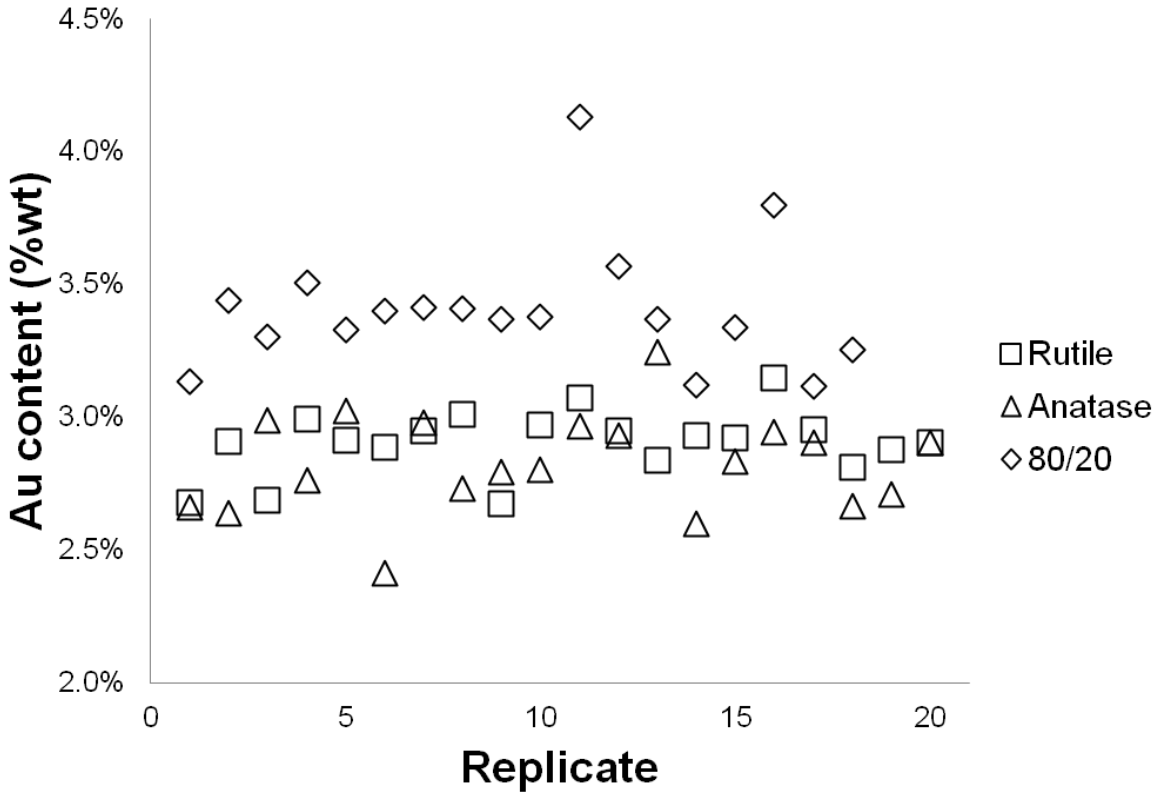

Supplementary Figure 6 individual results for 20 clusters of custom designed surface modified TiO_2 NP, ordered by intensity (lowest to highest, right to left)
